# Similar Effects for Resting State and Unconscious Thought: Both Solve Multi-attribute Choices Better Than Conscious Thought

**DOI:** 10.3389/fpsyg.2018.01360

**Published:** 2018-08-07

**Authors:** Fengpei Hu, Xiang Yu, Huadong Chu, Lei Zhao, Uyi Jude, Tao Jiang

**Affiliations:** ^1^College of Economics and Management, Zhejiang University of Technology, Hangzhou, China; ^2^Siyang Hospital of Traditional Chinese Medicine, Jiangsu, China; ^3^Zhijiang College of Zhejiang University of Technology, Shaoxing, China

**Keywords:** unconscious thought effect, job decision, resting state, thinking mode, decision performance

## Abstract

When people have headaches, they put their work aside and do other things. When they return, their decisions may be better, resulting in more satisfaction than if they had contemplated their choices consciously. Researchers have proposed the “deliberation-without-attention” hypothesis to discover whether it is always advantageous to engage in conscious deliberation before making a choice. Unconscious thinking can optimize people’s behavioral decision-making in a complex environment and improve their satisfaction with their choices. As previous studies have not used a resting state (RS), another kind of unconscious thinking, this paper adds the RS to unconscious thinking during distracting tasks, unconscious and conscious joint thinking, and conscious thinking conditions, to study the unconscious thought effect and decision-making performance in four different thinking modes. We performed three experiments involving a choice of jobs, using two ways of presenting information, to check the unconscious effect and compare the decision-making performance of different thinking patterns. The results show that RS and unconscious thinking have similar effects, while people’s decision-making performance differs in different thinking modes.

## Introduction

People face many difficult decisions, including how to find a good job, deal with leadership relationships, buy complex products, and achieve higher learning efficiency. When people buy complex products, people assigned to an unconscious thinking group have been shown to experience higher satisfaction than people assigned to a conscious thinking group. On the other hand, when buying simple products, the conscious thinking group experiences higher satisfaction than the unconscious thinking group (Dijksterhuis et al., 2006). Through such experiments, Dijksterhuis et al. (2006) proposed the “deliberation-without-attention” hypothesis, which states that, when target task processing is interrupted by an interference task, subjects enter a state of unconscious thought. Unconscious thought can optimize decision-making in complex situations and improve satisfaction with one’s choices. Through a large number of experiments, Dijksterhuis and Nordgren (2006) arrived at six principles of unconscious thought theory (the Unconscious-Thought, Capacity, Bottom-Up-Versus-Top-Down, Weighting, Rule, and Convergence-Versus-Divergence Principles), which explain the unconscious thought effect. Decision theorists have long recognized that people are constrained in their capacity to make decisions (Simon, 1955; Tversky and Kahneman, 1974; Bettman et al., 1998; Kahneman, 2003). As Wilson and Schooler (1991) made clear, a low capacity for conscious thinking leads to mistakes in decision-making. However, the capacity for unconscious thinking is not limited; in fact, it is very large.

The unconscious thought effect is a very interesting phenomenon that may be different from the processes we use to consciously make a decision. Various laboratories around the world are constantly studying this phenomenon; as they have not received exactly the same experimental results, there are still many controversies. Mcmahon et al. (2011) discussed the effect of the degree of difficulty of distracting tasks on unconscious thinking; this study found that simple distracting tasks, such as simply listening to music or searching for a letter, also supported an unconscious thinking mode (compared with word formation task), resulting in significantly better decision quality than that achieved by an immediate decision-making group engaged in conscious thinking. Several researchers have added a thinking mode: at the 4-min thinking stage, participants engage in conscious and unconscious thinking in a fixed order, with the first 2 min used for conscious thinking and the next 2 min for unconscious thinking, to determine decision quality (Nordgren et al., 2011). Results showed that decisions made through unconscious thinking were better than those made through immediate decision-making or conscious thinking, although the combination of thinking modes showed significantly better performance than unconscious thinking alone. However, Acker (2008) used the same experimental procedure as Dijksterhuis et al. (2006), the only changes being to translate the materials into English, and to use English-speaking subjects. As the unconscious thought effect was not reproduced, the findings did not show whether unconscious thinking in complex decision situations could help participants make better decisions than using conscious thinking. In addition, there was no significant difference in decision performance between unconscious and conscious thinking. In other words, unconscious thinking could not effectively improve decision performance (Newell et al., 2009). Mamede et al. (2010) divided subjects into an experienced group and a novice group; the results showed that the novice group showed an unconscious thinking effect when carrying out simple tasks, but this was not found under complex conditions. This study did not confirm the premise that the unconscious thought effect must occur in a complex decision situation. Researchers have also found that a significantly higher percentage of participants in an unconscious thinking group, as opposed to a conscious thinking non-information group, selected the highest lottery value. However, no significant difference was found between an unconscious group and a conscious information group. The results of these two experiments show that there is an unconscious effect, but do not support the capacity and weight principles of unconscious theory (Ashby et al., 2011). By contrast, the present study found that, no matter whether information was presented piece-by-piece or in lists, decision performance in a resting state (RS) and in an unconscious thinking state was significantly higher than decision performance in a conscious thinking state.

In addressing the unconscious thinking effect controversy, some scholars have studied unconsciousness from the perspective of cognitive neuroscience. Raichle et al. (2001) studied the RS using PET, and found higher activation in certain regions of the brain when it was in a RS but not during cognitive tasks. These regions have been named the default mode network (DMN); they mainly include the posterior cingulate cortex/posterior cortex, the anterior cingulate gyrus, precuneus, and medial prefrontal cortex. The DMN is the center of the brain’s functional linkage system, which maintains brain function activities in the RS and maintains close relationships with scene memory extraction, self-awareness monitoring, and other cognitive emotional processes. In addition, Buckner and Vincent (2007) and Buckner et al. (2008, 2009) explored whether brain activity in the RS can be considered as a baseline level. These findings suggest that observed neural responses can be approximated as a linear superposition of task-induced neural activity and persistent spontaneous activity, which means that spontaneous brain activity in the RS still exists in a task state. The brain not only does not stop working, but it also acts continuously in the RS, when there is no external task scenario. This condition is similar to the condition of unconscious thought with a distracting task because the RS can be understood as being similar to one in which attention has been diverted from the decision task by cognitive emotional processes subserved by the DMN.

The present study explores the effect of a RS on decision-making using a job decision task, both in a complex piece-by-piece information presentation condition and a simple listed-information presentation condition. Based on the theory of unconscious thinking and neuroimaging studies of the RS, we predicted that the RS and unconscious thinking will have a similar effect, both in the complex information presentation and simple listed-information presentation conditions. We therefore carried out three experiments to test this hypothesis. Experiment 1 examined decision-making performance resulting from the following four thinking patterns: RS, unconscious thought under the distraction task (UTDT), integration of conscious and unconscious thought (ICUT), and conscious thought (CT), in a complex information presentation condition. Experiment 2 examined decision-making performance resulting from these four thinking patterns in a simple listed-information presentation condition. In Experiment 3, we added an immediate decision group, to further explore the unconscious thought effects.

## Experiment 1

### Methods

#### Participants and Experimental Design

One hundred and twenty subjects were recruited from Zhejiang Sci-Tech University and Zhejiang University of Technology to participate in the experiment. All subjects were between the ages of 18 and 25 years, right-handed, and proficient in using computers. They received certain rewards after completing the experiment.

The experiment adopted a single-factor design, with a total of four conditions (four thinking modes): RS (12 male, 18 female), UTDT (16 male, 14 female), ICUT (14 male, 16 female), and CT (14 male, 16 female). As in Dijksterhuis et al. (2006), subjects were asked to choose the best out of four jobs and to rate their attitudes toward all of the jobs using a 30-point scale ranging from 1 to 30. The dependent variable was accuracy in choosing the best job and the differential attitude score was the difference between the best and the worst job.

### Materials

The experimental materials were based on Dijksterhuis et al. (2006). Using literature searches and a graduate career survey report (network of excellence), we summarized and obtained 20 dimensions of work that students would have to take into account when making job decisions (Fan, 2007, Unpublished; Zhou, 2009, Unpublished). We asked the subjects to score the importance of these 20 dimensions using a 7-point scale. After ranking the rating scores from the highest to the lowest, we chose the first 12 dimensions to describe four jobs separately, resulting in a total of 48 pieces of information. Using the analysis (Rey et al., 2009) of dimensions in Dijksterhuis et al.’s (2006) study, we assigned a positive or negative valence to the 12 dimensions, creating four jobs containing different pieces of information: the best job consisted of 75% positive features (nine attributes) and 25% negative features (three attributes); two medium jobs with 50% positive features (six attributes) and 50% negative features (six attributes); the worst job consisted of 25% positive features (three attributes) and 75% negative features (nine attributes). Consistent with previous studies, in relation to the top 3 most important dimensions, the best job was positive and the worst job was negative; the weighted sum of the importance of the best job was far higher than that of the worst job. The experimental materials (four multi-attribute jobs) are included in the Supplementary Material.

### Ethics Statement

All participants provided written informed consent before participating in the experiments. The participants were reminded of their right to discontinue participation at any time. The Research Ethics Board of Zhejiang Sci-Tech University and Zhejiang University of Technology approved all procedures.

### Procedures

**FIGURE 1:**
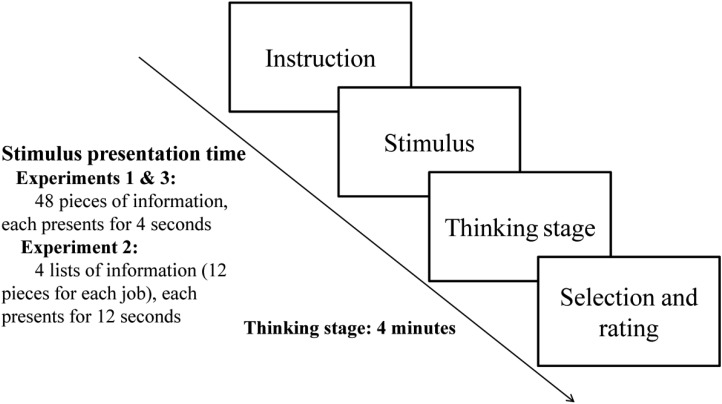
The experimental research procedure; except in Experiment 3, the immediate decision condition, subjects made their decisions right after the presentation of information.

The experimental procedures were written using E-Prime 2.0. Our procedure can be divided into four stages: the introduction stage (instruction), the information presentation stage (stimulus presentation), the thinking stage, and the selection and rating stage, as shown in **Figure 1**.

During the introduction stage, subjects were instructed that they would see information about four jobs and were asked to form an overall impression of these jobs. In addition, they were told that they would be required to choose the best job and to rate the jobs at the end of the experiment.

In the information presentation stage, the information was presented in the center of the computer screen. There was a total of 48 pieces of information (12 pieces for each job). All of these were randomized; only one piece of information was shown each time and the presentation lasted for 4 s.

The thinking stage lasted for 4 min. After the experimenter told the subjects to complete the target task, the subjects pressed a key to enter one of the conditions. There were four conditions:

(1) RS: Subjects were asked to relax, close their eyes, and try not to think about the target task—to be quiet and rest for 4 min.
(2) UTDT: To complete a 4-min anagram. For the anagram, English words were selected from junior high school vocabulary lists through a preliminary test and subject feedback. The first letter was fixed, and subjects were asked to arrange the remaining letters to form a word. For example, subjects given the letters “v r y e” would have to form the word “very.” The next trial would begin after the word was completed or after 45 s. The difficulty and number of words were chosen to ensure that the subjects were distracted for 4 min.
(3) ICUT: Subjects were asked to think carefully about the four jobs for 2 min and then to do the anagram for 2 min. This condition was based on Nordgren et al. (2011).
(4) CT: For 4 min, the subjects were asked to think carefully about the four jobs, using the information just presented.

At the selection and rating stage, subjects were asked to choose the best job out of the 4. Then they were asked to rate their attitudes toward the four jobs, using a 30-point scale (from 1 to 30).

### Data Analysis

We began by calculating the proportion of subjects who chose the best job; this represented the level of accuracy. This value was then used to index the quality of decision-making. To index each participant’s ability to tell the best from the worst job, we also calculated the differential attitude score between the best and the worst job (the attitude score for the best job minus the attitude score for the worst job). The differential attitude scores range from -29 to 29; the higher the score, the stronger the ability to distinguish, and the higher the quality of decision-making. Just as Dijksterhuis (2004) pointed out, recognizing the best alternative is obviously important, but recognizing and rejecting a particularly bad alternative is just as important in many situations. Furthermore, from a practical point of view, a score that shows the difference between two opposing attitudes may be more sensitive, providing more scope for discrimination than a non-parametric test, such as accuracy in choosing the best job.

### Results

#### Accuracy in Choosing the Best Job

**FIGURE 2:**
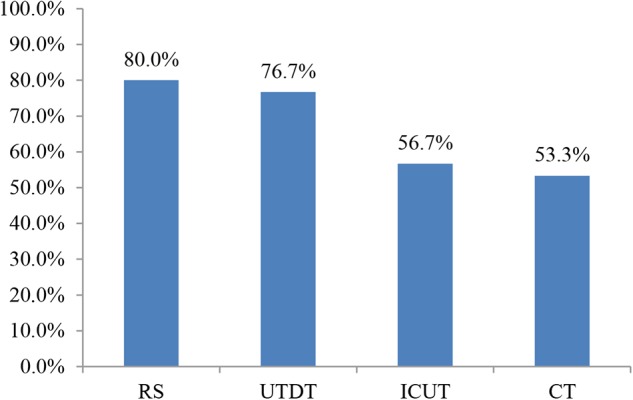
Accuracy in choosing the best job in different thinking modes when information was presented piece-by-piece in Experiment 1.

The participants’ accuracy in choosing the best job under each of the four conditions is shown in **Figure 2**. Accuracy was highest in the RS condition at 80% and lowest in the CT condition at 53.33%. Accuracy was higher in the UTDT condition (76.67%) than in the CT condition (53.33%). This showed the trend of the unconscious thought effect: in a complex decision-making situation, participants in the unconscious thought group performed better than those in the CT group. A chi-square test was carried out on the four thinking conditions in terms of accuracy. The results were as follows: χ^2^(3, 120) = 7.5, *p* = 0.058; this indicates that when information was presented item by item, there was a marginally significant difference in accuracy between the four thinking modes.

#### Differential Attitude Scores Under Different Thinking Modes

**FIGURE 3:**
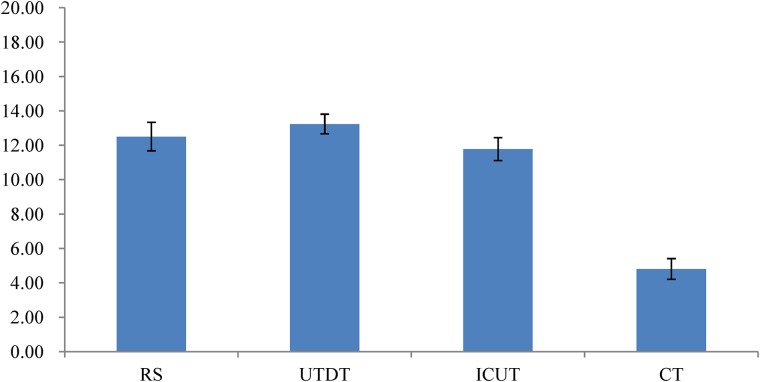
The mean of the differential attitude scores under different thinking modes in Experiment 1. Higher scores indicate larger attitude scores. Error bars indicate the SE of the differential attitude scores.

The result of a one-way analysis of variance (ANOVA) showed that when information was presented piece-by-piece, the differential attitude scores were significantly different in the four thinking modes, *F*(3,116) = 8.345, *p* < 0.01. This suggests that there was a significant difference between the four thinking conditions (RS, UTDT, ICUT, and CT) in participants’ ability to distinguish between the best and the worst job (**Figure 3**).

The Bonferroni *post hoc* comparisons of differential attitude scores under four thinking modes showed that the differential attitude scores under the UTDT condition (*M* = 13.23, *SE* = 1.13) were significantly higher than those under the CT condition (*M* = 4.8, *SE* = 1.2), *p* < 0.01. The differential attitude scores under the RS condition (*M* = 12.5, *SE* = 1.66) were also significantly higher than those under the CT condition, *p* < 0.01, while the differential attitude scores under the ICUT condition (*M* = 11.77, *SE* = 1.34) were significantly higher than those under the CT condition, *p* < 0.01. These results suggest that the subjects’ ability to distinguish between the best and the worst job under the RS and UTDT conditions was significantly higher than under the CT condition.

### Discussion

The differences in attitude scores showed that the participants’ decision-making performance was significantly higher in the unconscious thinking and RS conditions than in the conscious thinking condition. This finding verifies the unconscious thought effect. This is further supported by a marginally significant difference in best job accuracy between the four thinking conditions. These results are also consistent with those of previous studies (Dijksterhuis, 2004; Dijksterhuis et al., 2006). The results suggest that a RS and unconsciousness have a similar effect on making multi-attribute choices following a complex information presentation. To further test the effect of a RS, we conducted Experiment 2, which tested whether RS could produce a similar effect to UTDT in a simple information presentation condition.

## Experiment 2

### Methods

#### Participants and Experimental Design

One hundred and seventy subjects were recruited from Zhejiang Sci-Tech University and Zhejiang University of Technology to participate in the experiment. All subjects were between the ages of 18 and 25 years, right-handed, and proficient in using computers. They received certain rewards after completing the experiment. The experiment adopted a single-factor design and a total of four conditions: RS (17 male, 26 female), UTDT (16 male, 26 female), ICUT (19 male, 24 female), and CT (17 male, 25 female). The dependent variables were the same as in Experiment 1.

### Materials and Procedures

The materials and procedures were the same as in Experiment 1. The only difference was the method of presenting information about the four jobs. In Experiment 2, information about each job was presented in the form of a list. Twelve pieces of information about each job were listed on one page, resulting in a total of four lists, which were randomized and presented during the information presentation stage. The presentation time for each list was 12 s.

### Results

#### Accuracy in Choosing the Best Job

**FIGURE 4:**
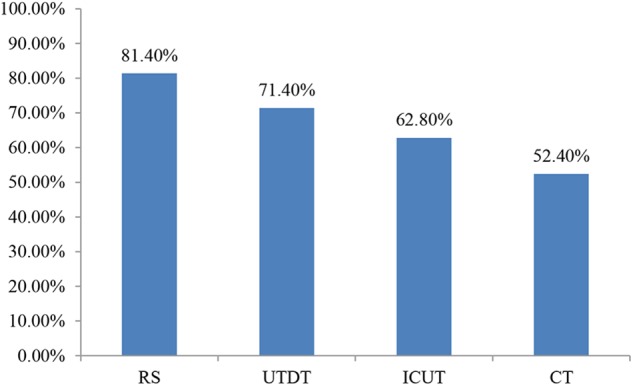
Accuracy in choosing the best job under different thinking modes when information was presented in lists in Experiment 2.

The accuracy in choosing the best job is shown in **Figure 4**. The accuracy of UTDT was 71.4%; CT had the lowest level of accuracy, at 52.4%. This showed the trend of the unconscious thought effect. As in Experiment 1, the accuracy of the RS condition (81.4%) was higher than that of the CT condition (52.4%). A chi-square test was carried out on the four thinking conditions in terms of accuracy and the result was χ^2^(3, 170) = 8.815, *p* < 0.05, suggesting that when information was presented in lists, the participants’ accuracy in choosing the best job was significantly different between the four thinking modes.

The *post hoc* comparison of best job accuracy in the four thinking modes showed that when information was presented in lists, the accuracy rate was significantly higher under the RS condition than under the CT condition, χ^2^(1, 85) = 8.097, *p* < 0.01. This suggests that participants’ accuracy in choosing the best job was significantly higher under the RS condition than under the CT condition. We found no significant difference in the accuracy rate between the other thinking modes, which were *p*s > 0.05.

#### The Differential Attitude Scores Under Different Thinking Modes

**FIGURE 5:**
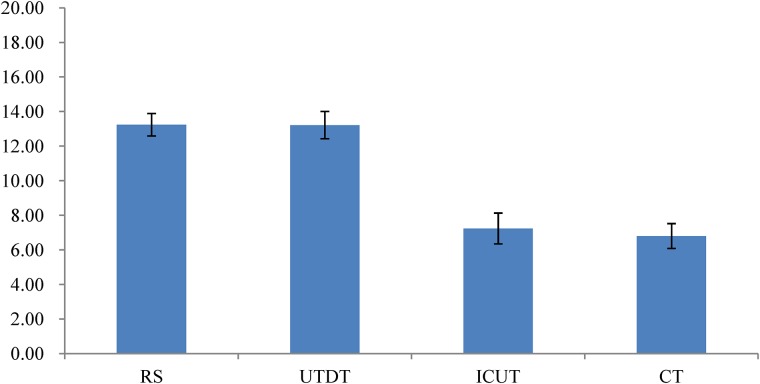
The mean of the differential attitude scores under different thinking modes in Experiment 2. Higher scores indicate larger attitude scores. Error bars indicate the SE of the differential attitude scores.

The results of a one-way ANOVA of the differential attitude scores under four thinking conditions showed that when information was presented in lists, the differential attitude scores were significantly different between the four thinking modes: *F*(3,166) = 5.479, *p* < 0.05. This finding indicates a significant difference in participants’ ability to distinguish between the best and the worst job between the four thinking modes (**Figure 5**).

The Bonferroni *post hoc* comparisons of the differential attitude scores under the four thinking modes showed that the differential attitude scores were significantly higher under the RS condition (*M* = 13.23, *SE* = 1.29) than under the CT condition (*M* = 6.79, *SE* = 1.44), *p* < 0.05; the differential attitude scores were also significantly higher under the UTDT condition (*M* = 13.21, *SE* = 1.58) than under the CT condition, *p* < 0.05. The differential attitude scores were significantly higher under the UTDT and RS conditions than under the ICUT condition (*M* = 7.23, *SE* = 1.78); both had *p*s < 0.05. We did not find a significant difference in differential attitude scores between the UTDT and RS conditions: *p* > 0.05. These results suggested that the subjects’ ability to distinguish between the best and worst jobs was significantly higher under the RS and UTDT conditions than under the CT condition.

### Discussion

The differential attitude scores in a RS were significantly higher than the scores in either a joint thinking or a conscious thinking state. In other words, the subjects’ ability to distinguish between the best and worst job was significantly higher in a RS than when they were jointly or consciously thinking. These findings confirm that people in a RS are better able to make multi-attribute choices than people using CT when information is presented as a simple list. At the same time, the participants’ attitude scores in the unconscious thinking condition were significantly higher than their scores in a conscious thinking condition. This result is the same result found in relation to a piece-by-piece presentation of information (Experiment 1). The results are consistent with Dijksterhuis (2004), who also used a simple presentation.

## Experiment 3

One may argue whether there is really a benefit to “unconscious thought” in a decision-making context, or whether these findings simply reflect memory distortion during information retrieval in CT. For instance, as mentioned above, Ashby et al. (2011) found that participants who deliberated on the information either equaled or outperformed those who processed the information using unconscious thought when choosing the best gamble. In addition, there was no difference between the unconscious thought and immediate decision groups. However, the gambling situation they used was based on abstract outcomes and probabilities, which assumes that individuals will use weights (probabilities) provided, rather than developed over time. For this reason, these findings may not suit the present situation. To further verify our results, we conducted a new experiment using four groups (UTDT, RS, CT, and immediate decision-making [I]).

### Methods

One hundred and twenty-eight subjects were recruited from Zhejiang University of Technology to participate in the experiment. All subjects were between the ages of 18 and 25 years, right-handed, and proficient in using computers. They received certain rewards for completing the experiment. The experiment adopted a single factor design and a total of four conditions: RS (19 male, 13 female), UTDT (9 male, 23 female), I (12 male, 20 female), and CT (9 male, 23 female). The dependent variables were the same as in Experiment 1.

### Materials and Procedures

The materials and procedures were the same as in Experiment 1. The only difference was that we added an immediate decision condition and removed the ICUT condition. In the immediate decision group, subjects selected the best job and scored different jobs immediately after seeing a total of 48 pieces of information presented piece-by-piece.

### Results

#### Accuracy in Choosing the Best Job

**FIGURE 6:**
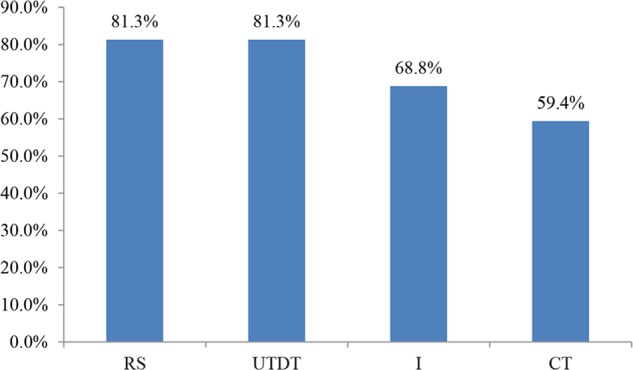
Accuracy in choosing the best job under different thinking modes when information was presented piece-by-piece in Experiment 3.

The participants’ accuracy in choosing the best job under the four conditions is shown in **Figure 6**. Unfortunately, the results did not replicate the findings of Experiments 1 and 2. The chi-square test showed no significant differences between the four conditions, χ^2^(3, 128) = 5.466, *p* = 0.141, suggesting that unconscious thought/RS did not help subjects choose the best job when the information was presented piece-by-piece, in comparison to immediate decision-making. For this reason, the “memory distortion” account cannot be ruled out.

#### The Differential Attitude Scores Under Different Thinking Modes

**FIGURE 7:**
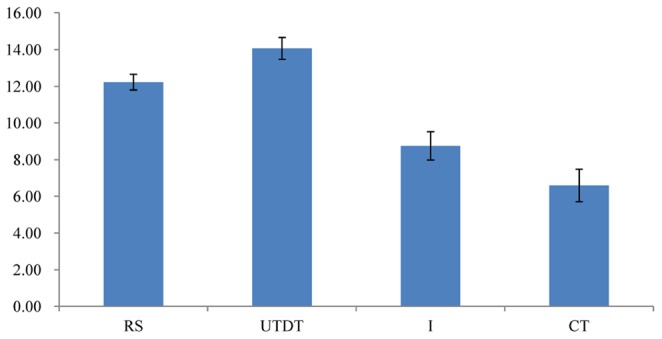
The mean of the differential attitude scores in different thinking modes in Experiment 3. Higher scores indicate larger attitude scores. The error bars indicate the SE of the differential attitude scores.

The result of a one-way ANOVA of the participants’ differential attitude scores under four thinking conditions showed that when information was presented piece-by-piece, the differential attitude scores were significantly different between the four thinking modes, *F*(3,166) = 5.884, *p* < 0.01. This suggests a significant difference in the ability to distinguish between the best and worst jobs in the four thinking modes (**Figure 7**).

The Bonferroni *post hoc* comparisons of differential attitude scores under four thinking modes showed that the differential attitude scores under the UTDT (*M* = 14.06, *SE* = 1.2) and RS (*M* = 12.22, *SE* = 0.85) conditions were significantly higher than those in the CT (*M* = 6.59, *SE* = 1.77) condition; in both cases, *p*s < 0.05. The difference between the UTDT and RS conditions was nonsignificant, *p* > 0.05. The difference between the CT and I (*M* = 8.75, *SE* = 1.55) conditions was also nonsignificant, *p* > 0.05. More importantly, the differential attitude scores under the UTDT condition were significantly higher than those under the I condition, at *p* < 0.05. The difference between the RS and I conditions was not significant, *p* > 0.05. These results, especially the difference between the UTDT and I conditions, showed that unconscious thought had some benefit in the decision-making process and that “memory distortion” alone cannot fully account for the effects.

### Discussion

In the current experiment, we introduced the immediate decision condition to test the “memory distortion” explanation of unconscious thought effects. The participants’ accuracy in choosing the best job showed no difference among the UTDT/RS and I conditions, suggesting that the information retrieval process could indeed cause some distortion during CT, impacting decision performance. For this reason, the “memory distortion” explanation cannot be ruled out. However, we did find a significant difference in the differential attitude scores: the participants were more able to distinguish between the best and worst jobs in the UTDT condition than in the I condition. This result suggests that unconscious thought does offer some benefit and may reflect an intermediate process before a decision is reached.

## General Discussion

Based on unconscious thinking theory and RS neuroimaging studies, this paper shows that the RS and unconscious thinking have a similar unconscious thought effects in the context of job multi-attribute decision-making: both allow subjects to make more accurate multi-attribute choices than CT. This effect was seen in both complex and simple information presentation conditions.

Nordgren et al. (2011) found that unconscious thinkers tended to choose the best apartment more often than conscious thinkers. The present study also found that decision performance was better in a state of unconscious thinking than in a state of CT. In line with previous studies on the unconscious effect (Dijksterhuis, 2004), the rates of correct choices made during unconscious thinking and the RS were higher than that of correct choices made during CT in Experiment 1. This finding indicated a trend for unconscious thought to be better than conscious thinking for solving complex problems. At the same time, subjects in the RS were distracted from the main decision task by cognitive emotional processes, subserved by the DMN. Unconscious thinking and the RS should therefore draw on similar unconscious thinking resources. The results of Experiment 1 found no difference between a RS and unconscious thinking when information was presented piece-by-piece. The participants’ decision performance in a RS and during unconscious thinking was significantly higher than their performance while engaging in conscious thinking, which confirmed our hypothesis.

A large meta-analysis sample has shown that the way in which information is presented can have a significant effect on the unconscious thinking effect (Acker, 2008; Strick et al., 2011). In addition, Dijksterhuis (2004) argued that presenting information in a list reduces decision-making difficulty and improves decision performance during conscious thinking. At the same time, researchers have explained that the unconscious effect is more obvious during a complex task and conscious thinking improves during simple tasks. The present study likewise found an unconscious thought effect when information was presented piece-by-piece in Experiment 1. When information was presented in lists, the unconscious thought effect also appeared in Experiment 2.

Our research found a marginally significant difference in the correct rate of selection between the four conditions in Experiment 1. However, the difference between the differential attitude scores was significant. This reflected the fact that participant scores were more sensitive than the correct rate and could therefore more accurately reflect the participants’ attitude to work under different conditions. In Experiment 2, when the information was presented in lists, there was a significant difference in the correct rate in the four thinking modes. This shows that the unconscious thought effect had a more significant effect on correct choices when information was presented in a list format.

Whether there is really a benefit to “unconscious thought” or these findings just reflect memory distortion during information retrieval in CT remains debatable. Ashby et al. (2011) found that participants who deliberated or made immediate decisions performed equally well or even better than unconscious thinkers when choosing the best gamble; this finding suggests the existence of a memory distortion. The present results, which found no difference between the unconscious thought/RS condition and the I condition for choosing the best job (Experiment 3), also support this finding. However, we did find a significant difference in the subjects’ differential attitude scores. The participants’ ability to distinguish between the best and worst jobs was higher in the UTDT condition than in the I condition, suggesting that unconscious thought does offer some benefit. This result is consistent with other studies that have used similar tasks (apartment and roommate selection, e.g., Dijksterhuis, 2004). Considering that recognizing and rejecting a particularly bad alternative is just as important as identifying the best one in many situations—and also that difference attitude scores may be more sensitive and able to discriminate than non-parametric tests (accuracy in choosing the best job), we can cautiously conclude that unconscious thought offers some benefit to the decision-making process, while “memory distortion” alone cannot fully account for the effects, at least in the present job selection scenario. Of course, more studies with varied tasks and measurements are needed to further address this issue.

In a previous study, decision tasks were transformed into medical-closer diagnostic tasks that were closer to real world situations, and the unconscious thought effect was experimentally confirmed (Vries et al., 2010). The decision-making situation used in our study was the choice of work. This is a situation that many students approaching graduation must face: choosing suitable work that reflects their own conditions and circumstances. The subjects recruited in this study were actual university students, including freshmen. Freshmen and sophomore students who do not yet have clear career plans or ideas may not feel that finding a job is an important problem for them. The results of this study could have been different if the selected subjects were graduates looking for a job or students who already had jobs.

## Author Contributions

FH conceived and designed the experiments. XY and HC performed the experiments. XY, LZ, and TJ analyzed the data. XY, FH, and LZ interpreted the data. XY drafted the paper. XY, UJ, HC, and LZ revised the paper. All authors edited and/or approved the manuscript.

## Conflict of Interest Statement

The authors declare that the research was conducted in the absence of any commercial or financial relationships that could be construed as a potential conflict of interest.
