# Supplementary material for: Similar Effects for Resting State and Unconscious Thought: Both Solve Multi-attribute Choices Better Than Conscious Thought
Source: Front Psychol. 2018 Aug 7;9:1360. doi: 10.3389/fpsyg.2018.01360 (PMC6090893; doi:10.3389/fpsyg.2018.01360)
Supplement: Supplementary file 1 [file Table_1.pdf]

SUPPLEMENTARY MATERIAL:

job decision material

**Best work**	**Worst work**
Higher wages	Wage is low
Vacation more	Less vacation
Company system clear	Company system is not standardized
The working environment is noisy	The working environment is noisy
Located in the more developed areas	Located in more remote areas
No training	No training
The boss is kind and kind	The boss is rude and irritable
Work intensity	Work intensity is small
Accord with hobbies	Hobbies do not match
Have more subsidy	Have more subsidy
Package three meals accommodation	Package three meals accommodation
Fortune 500 companies	Small and medium enterprises

**Medium work 1**	**Medium work 2**
Higher wages	Wage is low
Less vacation	Vacation more
Company system clear	Company system is not standardized
The working environment is noisy	The working environment is clean and tidy
Located in the more developed areas	Located in more remote areas
Have regular training	Have regular training
The boss is kind and kind	The boss is rude and irritable
Work intensity is small	Work intensity
Hobbies do not match	Accord with hobbies
No subsidy	No subsidy
Three meals a day to take care of themselves	Package three meals accommodation
Small and medium enterprises	Fortune 500 companies
